# Antimicrobial activity of cefepime-tazobactam combination against extended spectrum beta-lactamase and/or AmpC beta-lactamase- producing gram-negative bacilli

**DOI:** 10.1186/s12879-024-09296-y

**Published:** 2024-04-23

**Authors:** Basma Ahmed Elawady, Noha Refaat Mahmoud, Hala El-Sayed Badawi, Azza Essam Eldin Badr, Noha Mahmoud Gohar

**Affiliations:** 1 Medical Microbiology and Immunology Department, Faculty of Medicine, Cairo University, Cairo, Egypt; 2 Medical Microbiology and Immunology, Theodor Bilharz Research Institute, Warraq Al Hadar, Egypt

**Keywords:** Cefepime-tazobactam, ESBL, AmpC, Gram-negative bacilli

## Abstract

**Background:**

The problem of resistance to beta-lactam antibiotics, which is caused by ESBL and AmpC β-lactamases, is getting worse globally. Infections caused by bacterial isolates harboring these enzymes are difficult to treat with carbapenems being the sole effective treatment option for such infections. The objective of this study was to determine the frequency of ESBLs and AmpC-producing Gram-negative bacilli isolated from clinical specimens and to evaluate the sensitivity of cefepime-tazobactam combination against them.

**Methods:**

This is an observational cross-sectional study carried out on 100 Gram-negative bacilli at Theodor Bilharz Research Institute Hospital during the period from February 2015 to January 2016. ESBL production was screened by using the disc diffusion test followed by confirmation by the combined disc confirmatory test, the screening for AmpC production was conducted using the cefoxitin disc test, which was subsequently confirmed by the AmpC disc test. Isolates confirmed positive for ESBL and/ or AmpC production were investigated for their susceptibility to antibiotics.

**Results:**

Among 100 Gram-negative bacilli, 44 isolates were confirmed as ESBL producers by the combined disc confirmatory test out of 56 isolates that tested positive for ESBL production through the disc diffusion test. The presence of AmpC production was assessed using the cefoxitin disc test, 32 isolates were screened to be AmpC producers, and the AmpC disc test confirmed AmpC production in 9 isolates of them. Using the Mast® D68C set, 32 isolates were ESBL producers, 3 were AmpC producers, and 4 isolates were ESBL/AmpC co-producers. The highest sensitivity was to cefepime-tazobactam (91.48%) followed by the carbapenems.

**Conclusion:**

Cefepime-tazobactam showed remarkable activity against ESBL and/or AmpC-producing Gram-negative bacilli and may be considered as a therapeutic alternative to carbapenems.

## Introduction

Drug resistance in Gram-negative bacilli is a significant global public health concern [1]. Gram-negative bacilli (GNB) such as *Klebsiella pneumoniae*, *Escherichia coli*, *Acinetobacter baumannii*, and *Pseudomonas aeruginosa* are some of the most significant bacteria causing nosocomial and community-acquired infections [2, 3].

Multidrug-resistant GNB infections are most frequently treated with beta-lactam antibiotics. However, the threat of beta-lactam antibiotic resistance is spreading globally as a result of the production of beta-lactamases [4]. According to Ambler categorization, the beta-lactamases can be categorized phenotypically into four classes (A-D) and functionally into three groups [5, 6].

A large majority of beta-lactam antibiotics, including penicillins, cephalosporins, and monobactams, can be hydrolyzed by extended-spectrum β-lactamases (ESBLs), with the exception of cephamycins and carbapenems, ESBLs can only be inhibited by beta-lactamase inhibitors like clavulanic acid, sulbactam and tazobactam [7].

AmpC β-lactamases, belonging to the class C category, hold clinical significance due to their ability to confer resistance in GNB against penicillin, cephalosporin, cephamycin, and monobactam. Unlike ESBL enzymes, AmpC β-lactamase activity remains unaffected by ESBL inhibitors [6].

Treatment options currently include beta-lactam/beta-lactamase inhibitor combinations, colistin, carbapenems, fosfomycin, and tigecycline [8, 9].

Cefepime is the fourth-generation cephalosporin and has an extended spectrum of activity against GNB. It lacks activity against ESBLs but it is stable against AmpC, while tazobactam is active against ESBLs. As a result, it is anticipated that Enterobacterales will become more susceptible when cefepime and tazobactam are combined [10].

Obtaining adequate knowledge regarding the magnitude and scope of ESBLs and AmpC production in GNB is vital for implementing strategies that can effectively reduce their transmission [4]. Only a limited number of studies are available for cefepime-tazobactam combination so, we aimed to determine the frequency of ESBLs and AmpC-producing GNB isolated from clinical specimens and to evaluate the sensitivity of cefepime-tazobactam combination against ESBL- and/or AmpC-producing GNB.

## Materials and methods

This is an observational cross-sectional study carried out on 100 GNB isolates from various clinical specimens (urine, pus, sputum, blood, and ascitic fluid). These isolates were obtained from outpatient clinics and patients who were hospitalized at Theodor Bilharz Research Institute (TBRI) Hospital during the period from February 2015 to January 2016. The study was approved by the Faculty of Medicine at Cairo University on 15/6/2015.

### Culture and identification

- Urine, sputum, and pus samples were directly plated onto MacConkey agar (Oxoid, UK). Furthermore, urine samples were cultured using CLED agar (Oxoid, UK). The cultured plates were then incubated aerobically at 37 °C to be inspected for growth after 18–24 h [11].
- Blood and ascitic fluid samples were inserted in Bactec blood culture bottles (Becton Dickinson International, Belgium) and put in a BACTEC 9010 device, followed up for a maximum of five days to detect a positive alarm signal in Bactec. Subcultures were subsequently done on MacConkey agar [11].
- All GNB growing on MacConkey agar and CLED agar were identified systematically using conventional biochemical reactions [11]. Bacterial isolates that were not conclusively identified to species level by the conventional biochemical reactions were tested using the analytical profile index API-20E for Enterobacterales and API-20NE for non- Enterobacterales (Bio-Mérieux, France).

### Detection of ESBL-producers among isolated GNB

1. ESBL production screening using disc diffusion method

The disc diffusion method was carried out on Mueller Hinton agar (MHA) (Oxoid, UK). The antibiotic discs (Oxoid, UK) used were: ceftazidime (CAZ, 30 µg), cefotaxime (CTX, 30 µg), ceftriaxone (CRO, 30 µg), cefpodoxime (CPO, 10 µg) and aztreonam (ATM, 30 µg). The inoculated MHA plates were incubated at 35 °C for 16–18 h. results were interpreted according to CLSI, (2015) [12].

2. Confirmation of ESBL production using combination disc diffusion method

Isolates that tested positive for ESBL production in the initial screening were further confirmed using the combination disc diffusion method. On MHA, ceftazidime discs (CAZ, 30 µg) alone, and ceftazidime discs plus clavulanate (CCAZ, 10 µg) were applied. An ESBL-producing bacterium was identified when there was a difference in diameter of 5 mm or greater between the antibiotic inhibitory zone alone and the combined disc with clavulanate [12].

### Detection of AmpC-producers among isolated GNB

1. Screening for AmpC production using Cefoxitin disc

Inhibitory zones less than 18 mm in diameter for cefoxitin disc (30 µg) indicate the possibility of AmpC production by the tested organisms [13].

2. Confirmation of AmpC production using AmpC disc test

A reference strain *E. coli* ATCC 25,922 (sensitive to cefoxitin, obtained from TBRI) was used. Testing and interpretation were carried out according to Singhal et al. [14]. A positive result was determined by the presence of an indentation or flattening of the cefoxitin inhibition zone near the test disc, indicating resistance. Conversely, a negative result showed an undistorted zone around the disc, indicating susceptibility.

### Mast® D68C ESBL and AmpC detection set

The isolates that showed positive results for ESBL and/or AmpC production in the screening tests were further analyzed using the Mast® D68C ESBL and AmpC detection set (Master Group, UK). Based on the manufacturer’s recommendations, testing and interpretation were carried out. *E. coli* ATCC 25,922 was used as a quality control strain.

### Testing susceptibility patterns of the isolated organisms

The Kirby-Bauer disc diffusion method was carried out for antimicrobial susceptibility testing for the confirmed positive ESBL and/or AmpC-producing-GNB isolates. The following antibiotic discs were tested: Cefepime (FEP, 30 µg), imipenem (IPM, 10 µg), ertapenem (ERT, 10 µg), meropenem (MEM, 10 µg), piperacillin-tazobactam (TPZ, 75/10µg), cefoperazone-sulbactam (CES, 75/30µg) and cefepime-tazobactam (CPT, 30/10µg) (Oxoid, UK). Antibiotics’ inhibitory zones were measured and the tested organism was reported as sensitive or resistant according to CLSI guidelines [12].

### Statistical analysis

SPSS statistical program version 16 was used to analyze the data. Frequency and percentage were used to express qualitative data. The accuracy of the test was assessed using specificity, sensitivity, positive and negative predictive values as measures.

## Results

Out of 100 GNB, 77 isolates were obtained from urine specimens, 10 isolates from pus specimens, 6 isolates from sputum, 5 isolates from blood, and 2 isolates from ascitic fluid.

The most commonly encountered organisms were *Escherichia coli* (*E. coli)* (51) followed by *Klebsiella pneumoniae* (*K. pneumoniae*) (32) and *Acinetobacter baumannii* (*A. baumannii*) (10), *Pseudomonas aeruginosa* (*P. aeruginosa*) *(3)*, *Enterobacter cloacae* (*E. cloacae*) (2), and *Providencia stuartii* (1), *Serratia marcescens* (*S. marcescens*) (1) isolate.

### Detection of ESBL-producers among isolated GNB

1. Screening for ESBL production

**Fig. 1:**
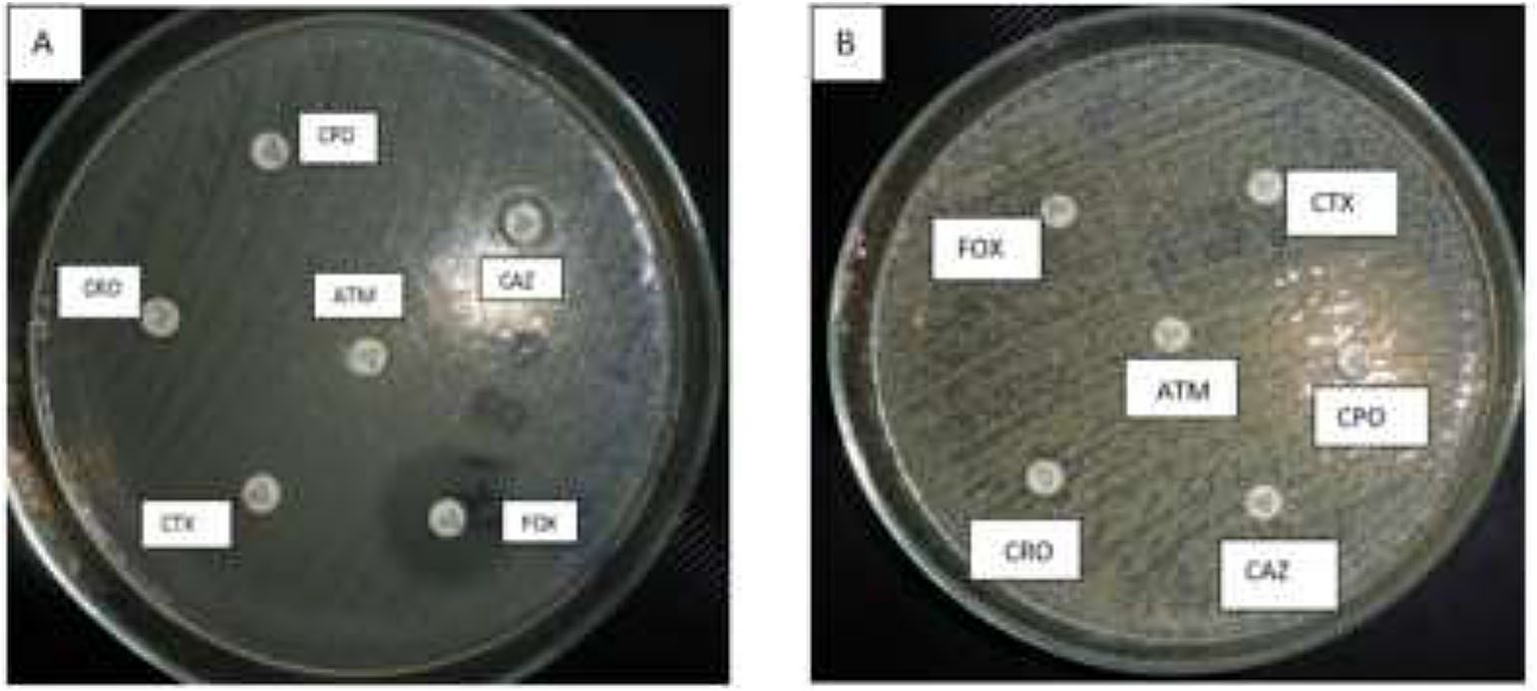
Screening for ESBL production by the disc diffusion test and screening for AmpC production by the cefoxitin disc test **(A)**: An ESBL-producing *E. coli* isolate showing resistance to (CAZ), (CPO), (CTX), (ATM) and (CRO). The organism was sensitive to cefoxitin (FOX) indicating that the isolate is a non-AMPC-producer. **(B)**: An AMPC-producing *A. baumannii* isolate showing resistance to cefoxitin disc (FOX).

Out of the 100 GNB, 56 isolates were positive for the production of ESBL through the disc diffusion test (Fig. 1). These 56 isolates included 23 *K. pneumoniae*, 19 *E. coli*, 9 *A. baumannii*, 3 *P. aeruginosa*, and only one *E. cloacae* isolate and one *S. marcescens* isolate. ESBL was detected in 72% (23/32) of *K. pneumoniae* isolates, 37% (19/51) of *E. coli* isolates, and 90% (9/10) of *A. baumannii* isolates.

2. ESBL production confirmation

**Fig. 2:**
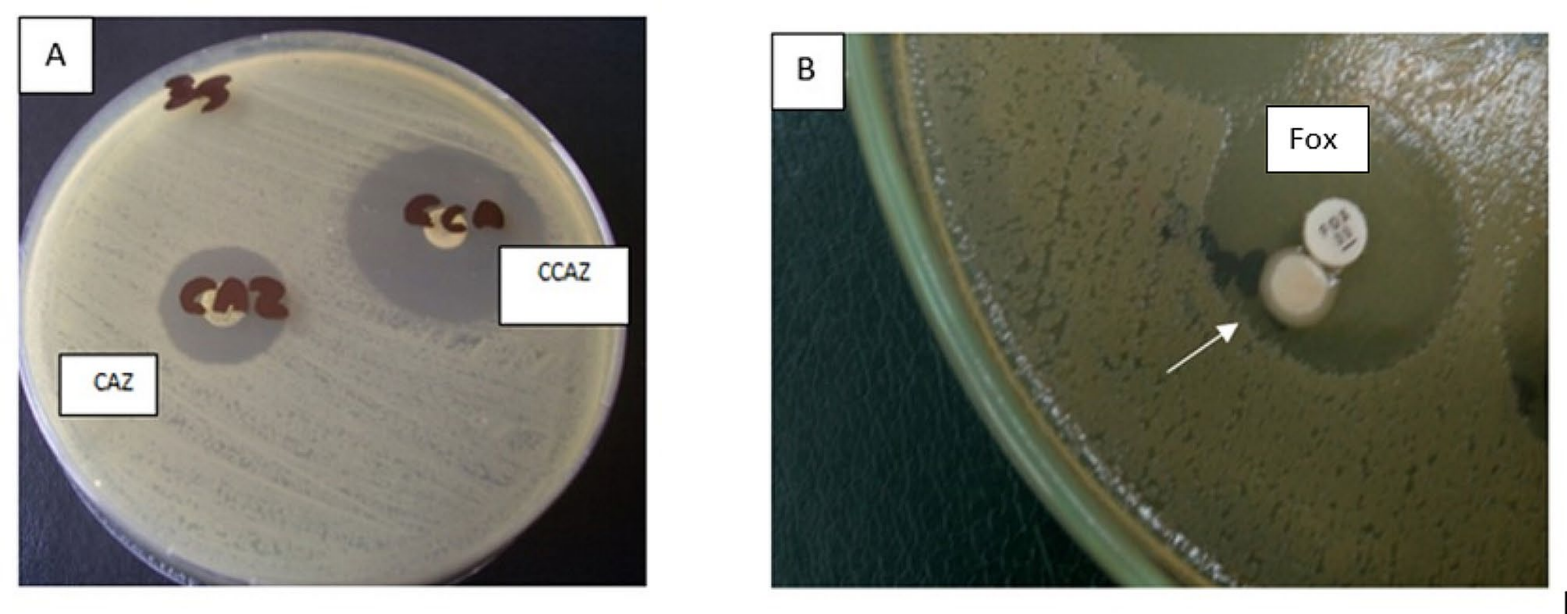
Confirmation of ESBL production by combined disc method and confirmation of AmpC production by by AmpC disc **(A)**: Confirmation of ESBL production by the combined disc method: an ESBL-producing *E. coli* isolate showing ˃ 5 mm difference in zone diameter between ceftazidime (CAZ) and ceftazidime plus clavulanic acid (CCAZ). **(B)**: Confirmation of AmpC production by AmpC disc in a *K. pneumoniae* isolate; there is flattening in the cefoxitin (FOX) inhibitory zone (arrow)

The combined disc confirmatory test was performed on the 56 isolates that tested positive for ESBL production through the disc diffusion test (Fig. 2). The combined disc test confirmed 44 isolates as ESBL-producers. The results were the same as those of the screening test except the 9 *A. baumannii* isolates and the 3 *P. aeruginosa* isolates which were non-ESBL producers by the combined disc test.

### Detection of AmpC-producers among isolated GNB

1. Screening for AmpC production

Out of 100 GNB, 32 isolates tested positive for AmpC production through cefoxitin disc test. These isolates were among those screened positive for ESBL production (Fig. 1). They included 12 *K. pneumoniae* (12/32; 37.5%), 8 *A. baumannii* (8/10; 80%), 7 *E. coli* (7/51; 13.7%), 3 *P. aeruginosa*, one *E. cloacae*, and one *S. marcescens*.

2. Confirmation of AmpC production

The 32 isolates that screened as AmpC producers were subjected to the AmpC disc test as a confirmatory method to confirm AmpC production (Fig. 2). The AmpC disc test confirmed 9 isolates as AmpC producers: 4 *K. pneumoniae*, 3 *A. baumannii*, one *E. coli*, and one *E. cloaca*. AmpC was detected in 30% (3/10) of *A. baumannii*, 12.5% (4/32) of *K. pneumoniae* isolates, and 2% (1/51) of *E. coli* isolates.

According to the confirmatory tests employed for ESBL or AmpC production, a total of 47 confirmed positive for ESBL and/or AmpC production in our study. 38 isolates proved to be ESBL-producers only (19 *K. pneumoniae*, 18 *E. coli*, and one *S. marcescens*), 3 AmpC-producers only (3 *A. baumannii*), and 6 isolates were producing both enzymes (4 *K. pneumoniae*, one *E. coli*, and one *E. cloaca*).

### Mast® D68C ESBL and AmpC detection set

**Fig. 3:**
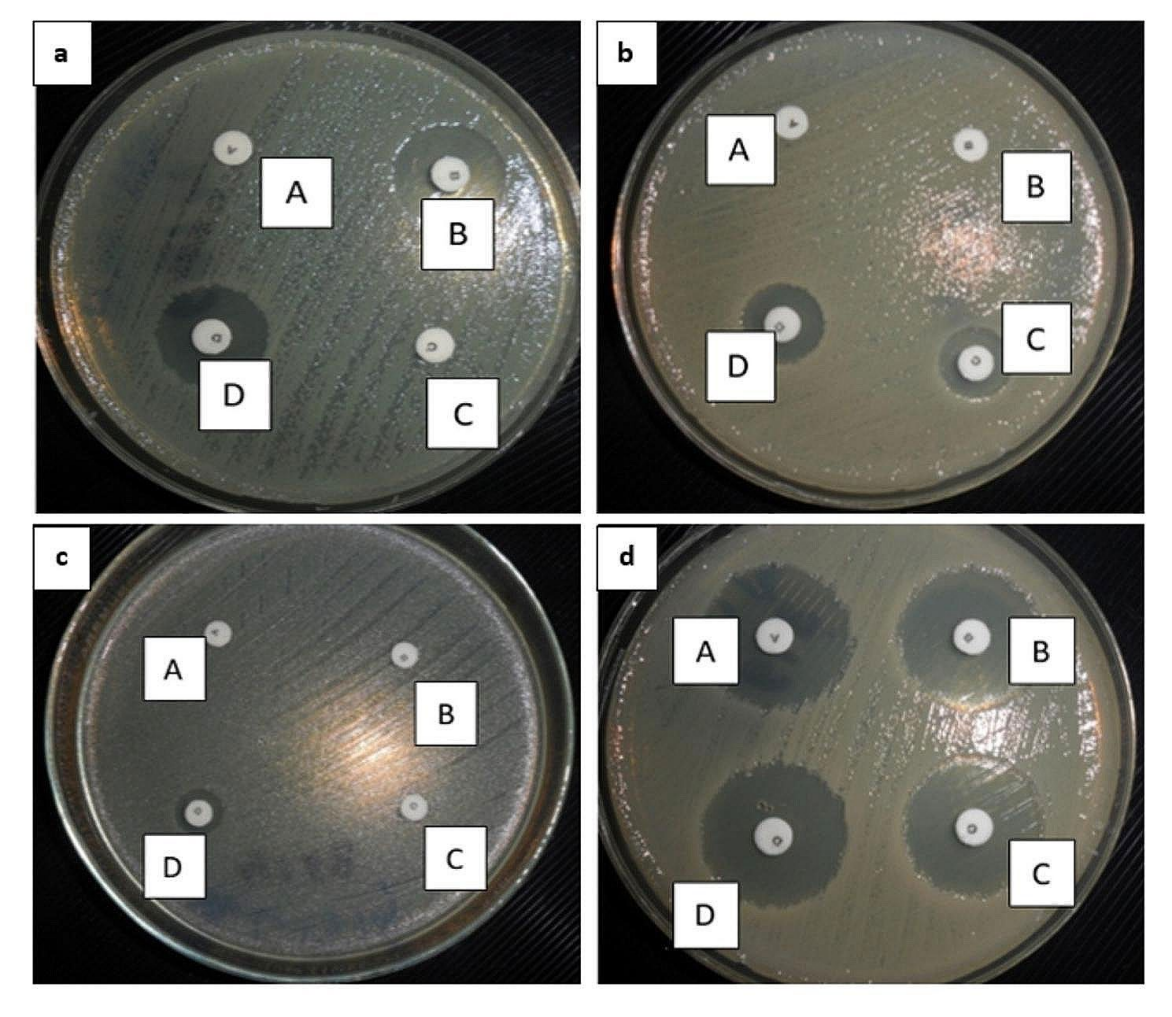
**Detection of ESBL and/or AmpC production using the Mast D68C** Disc A contained cefpodoxime (10 µg), Disc B contained cefpodoxime (10 µg) and an ESBL inhibitor, Disc C contained cefpodoxime (10 µg) and an AmpC inhibitor, Disc D contained cefpodoxime (10 µg) and both the AmpC and ESBL inhibitors **(a)**: An *E. coli* isolate showing ESBL production only: B - A and D - C ≥ 5 mm AND The difference between B & D and between A & C are < 4 mm **(b)**: A *K. pneumoniae* isolate showing AmpC production only: C - A and D - B are ≥ 5 mm AND The difference between A & B and between C & D are < 4 mm **(c)**: An *E. coli* isolate showing ESBL and AmpC co-production: D - C ≥ 5 mm AND The difference between A & B is < 4 mm **(d)**: A *K. pneumoniae* isolate showing negative result for both ESBL and AmpC production: A difference of ≤ 2 mm is present between all zones (A, B, C and D)

Testing by Mast® D68C set was performed on all 56 isolates screened positive for either ESBL and/or AmpC. The test demonstrated that 32 (57%) isolates were positive for ESBL production only and they were distributed as follows: 16 *E. coli*, 15 *K. pneumoniae*, and one *P. aeruginosa*. (Fig. 3, a), 3 (5%) isolates, consisting of 2 *A. baumannii* and one *K. pneumoniae* were positive for AmpC production only (Fig. 3, b), 4 (7%) isolates, including one *E. coli*, one *K. pneumoniae*, one *A. baumannii*, and one *E. cloacae* were ESBL/AmpC co-producers (Fig. 3, c), 17 (31%) isolates consisting of 6 *K. pneumoniae*, 2 *E. coli*, 6 *A. baumannii*, 2 *P. aeruginosa*, and *one S. marcescens* were negative for both enzymes (Fig. 3, d).

### Antibiotic susceptibility pattern of bacterial isolates

**Fig. 4:**
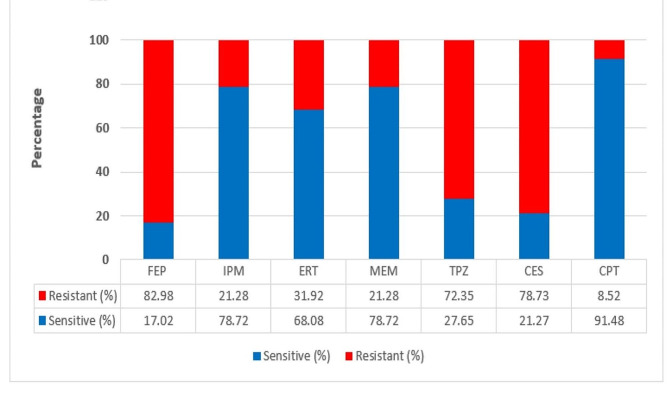
Antibiotic susceptibility patterns of the 47 bacterial isolates confirmed positive for ESBL and/or AmpC production

The highest sensitivity was to cefepime-tazobactam (91.48%) followed by the carbapenems. Sensitivity to cefepime alone was 17.02%; the addition of tazobactam raised the sensitivity of the isolates to the combination to 91.48%. One *K. pneumoniae* isolate (ESBL-producing) and one *A. baumannii* isolate (AmpC-producing) were sensitive only to cefepime-tazobactam combination. One *E. coli* isolate (ESBL-producing) was sensitive only to cefepime-tazobactam and imipenem. Two *K. pneumoniae* isolates (ESBL and AmpC-producing), one *E. coli* isolate (ESBL-producing), and one *A. baumannii* isolate (AmpC-producing) were resistant to the cefepime-tazobactam combination. All these isolates were resistant to all other antibiotics used in our study, except for one *K. pneumoniae* which was sensitive to imipenem and meropenem only. Other combinations were less active against different isolates (Fig. 4).

The susceptibility pattern of the 47 bacterial isolates to cefepime-tazobactam was illustrated in Table 1.

**Table 1:** Susceptibility patterns of ESBL/AmpC positive isolates (47) to cefepime-tazobactam

	Sensitive n (%)	Resistant n (%)
*E. coli (19)*	18 (94.73)	1 (5.27)
*K. pneumoniae (23)*	21 (91.30)	2 (8.70)
*A. baumannii (3)*	2 (66.66)	1 (33.34)
*E. cloacae (1)*	1	0
*S. marcescens (1)*	1	0

## Discussion

The rise of antimicrobial resistance poses a significant challenge to healthcare systems globally [15, 16]. Extended-spectrum beta-lactamase-producing GNB presents significant treatment challenges, leading to difficulties and failures [4]. In our study, 100 GNB isolates were assessed for ESBL and AmpC production by disc diffusion method and cefoxitin disc test, respectively. The positive ones were subjected to the confirmatory tests, combined disc (for ESBL) and AmpC disc test (for AmpC).

In this study, the percentage of confirmed ESBL producers among the 100 GNB isolates was 44%. This was relatively in agreement with previously reported studies in Egypt which was 53.3% [17], and 57.8% [18]. Other studies have reported higher rates of ESBL production in Egypt; Gharib et al. found a rate of 60.2% in a critical care center at Kasr Al-Ainy Hospital [19]. Amer et al. identified 67.26% of *E. coli* and *Klebsiella pneumoniae* isolates from Kasr Al-Ainy Hospitals were phenotypically positive for ESBLs [20]. On the other hand, lower rates were recorded by several previous Egyptian studies; 23.8%, 17%, and 38.8% [21–23]..

In our study, a higher percentage of ESBL production was observed among *Klebsiella pneumoniae.* (72%) *versus E. coli* (37%). Mohamed et al. found a higher ESBL production among *Klebsiella pneumoniae* (74.6%) than *E. coli* (69.6%) [24]. Our findings disagree with some Egyptian studies; these studies reported a higher percentage of ESBL production among *E. coli* compared to non-*E. coli* isolates [22, 25].

In this study, the percentage of isolates that were confirmed to produce AmpC enzyme was 9%. Oberoi et al. reported that 5.4% of GNB isolates were AmpC-producers [26]. A lower rate was recorded by another study which showed AmpC production in 2.6% of studied Enterobacterales isolates [27], whereas higher percentage rates of 28.3% and 19.5% were reported [28, 29]. Sultan et al. reported that 49% of GNB isolates were AmpC-producers [30]. The observed differences in prevalence rates could be attributed to various factors such as variations in the geographic regions, sample sizes, types of specimens analyzed, the specific species of bacteria isolated, study population, and the extent of antibiotic usage in different settings [4].

In this study, a higher percentage of AmpC production was observed among *A. baumannii* (30%), followed by *K. pneumoniae* (12.5%) than among *E. coli* isolates (2%). A similar finding was observed by Sultan et al. who reported that 44.4% of *A. baumannii* isolates were AmpC-producers, which was greater than the AmpC production rates of *E. coli* (35.5%) and *K. pneumoniae* (30.4%) [30]. Yilmaz et al. found that 10% of *K. pneumoniae* isolates were AmpC-producers and 0.9% in *E. coli* [31]. Other investigators found higher rates of AmpC production among *E. coli* (9% and 5.2%, respectively) [32, 33]. According to a study carried out by Salamat et al. *Enterobacter species* were the most common AmpC-producing isolates recovered from neonates with sepsis [34].

In most of the world, the production of AmpC is less frequent than ESBL enzymes. On the other hand, both enzymes could be found in one strain, which confers resistance to all β-lactams except cefepime and carbapenems [35].

According to the confirmatory tests employed for ESBL or AmpC production in our study, 12.8% were ESBL/AmpC co-producers. Tekele et al. showed that 3.6% of isolates produced both ESBL and AmpC enzymes [4]. Other studies conducted in Nigeria and South India found a rate of 6.04%, and 4.4% respectively [3, 36].

The Mast® D68C set is a simple phenotypic test used for easily identifying ESBLs [37]. In our study, testing by Mast® D68C set was performed on all 56 isolates screened positive for either ESBL and/or AmpC. The test demonstrated that 57% of the isolates were positive for ESBL production only, 5% were positive for AmpC production only, 7% were ESBL/AmpC co-producers, and 31% were negative for both enzymes.

These results were compared to those of another study using the same kit, the authors stated that the percentage of ESBL-producers was 65.8%, AmpC-producers was 2.6%, whereas 31.6% were neither ESBL nor AmpC-producers [27]. Using the same kit, Rizi et al. reported that 30% of isolates simultaneously exhibited ESBL and AmpC activity [38].

In this study, Mast® D68C set gave 81.8% sensitivity, 100% specificity, 100% PPV and 60% NPV in ESBL detection. A similar study reported 97.2% sensitivity, 88.8% specificity, 97.2% PPV, and 88.8% NPV [39].

In our study, The Mast® D68C set revealed 77.7% sensitivity, 100% specificity, 100% PPV and 92% NPV in AmpC detection. El Sayed et al. reported 60% sensitivity, 100% specificity, 100% PPV, and 66.7% NPV [39].

In our study, resistance to carbapenems ranged from 21.27% to imipenem and meropenem and 31.9% with ertapenem. Another study recorded a 20.9% resistance rate to imipenem [40]. Sultan et al. observed lower resistance rates to imipenem and meropenem (13.7, and 8.2%, respectively) among AmpC-producers isolates [30]. The least effective carbapenem evaluated in our investigation was ertapenem (68% sensitivity). This was in disagreement with Owusu et al. who showed that ertapenem was one of the most efficient antibiotics among the carbapenems studied [41].

A number of β-lactam/βlactamase inhibitor antibiotics have demonstrated synergistic effects against multidrug-resistant bacteria, such as ampicillin-sulbactam, amoxicillin-clavulanate, cefoperazone-sulbactam, piperacillin-tazobactam, and ceftazidime-avibactam [42].

In our study, the susceptibility pattern of the isolates was tested for cefoperazone-sulbactam and piperacillin-tazobactam. Most of our isolates were resistant to both combinations (72.35% and 78.73%, respectively). Sultan et al. reported a 97.3% resistance rate to piperacillin-tazobactam among AmpC-producer isolates [30].

Cefepime is known for its stability in the presence of AmpC enzymes, suggesting that its main vulnerability lies in protection against ESBLs. On the other hand, tazobactam exhibits greater activity in inhibiting ESBLs when compared to clavulanic acid and sulbactam [43].

To the best of our knowledge, a limited number of studies about cefepime-tazobactam combination (CPT) have been published till now, especially in Egypt. In this study, sensitivity to cefepime alone was 17.02%; the addition of tazobactam raised the sensitivity of the isolates to the combination to 91.48%. Ghafur et al. reported that the addition of tazobactam raised the sensitivity from 46.2 to 80.4% [40]. Other studies tested the sensitivity of ESBL-producers to CPT combination; Mudshingkar et al. reported that 94.1% of ESBL-producing isolates were sensitive to CPT [44]. Susan et al. reported lower sensitivity (73%) and concluded that the addition of tazobactam to cefepime raised the sensitivity of the isolates from 34.2 to 73% [45].

The sensitivity to CPT in our study was highest among *E. coli* isolates (94.7%) followed by *K. pneumoniae* (91.3%) and then *A. baumannii* (66.6%). These results were in relative agreement with another study which stated that 86.9%, 67.1%, and 25.8% of *E. coli, K. pneumoniae*, and *A. baumannii*, respectively, were sensitive to cefepime-tazobactam [45]. Another study revealed that CPT was highly effective against *E. coli, Enterobacter*, and *Proteus mirabilis*, on the other hand, it was not very efficient against *K. pneumoniae* (36% sensitivity), however, the authors stated that CPT performed better for all isolates than either cefepime or piperacillin-tazobactam administered alone [10].

According to Sader et al. cefepime-tazobactam inhibited 96.1% of *Enterobacter species* and 91.6% of *Pseudomonas aeruginosa* isolates, which was higher in its effectiveness than meropenem and piperacillin-tazobactam (79.2% sensitivity) [46]. Mushtaq et al. found that cefepime-tazobactam was widely effective against ESBL/AmpC-producing Enterobacterales, and they discovered that CPT had a spectrum that was greater than those of piperacillin-tazobactam and carbapenems [43].

Based on the results of the current study; we concluded that a high percentage of ESBL and AmpC production was reported among 100 GNB isolates. Our *In-vitro* susceptibility results suggested that the cefepime-tazobactam combination has excellent activity against ESBL and/or AmpC-producing GNB which can contribute to a decrease in the utilization of carbapenems and thus emergence of carbapenem resistance suggesting that this combination can act as a carbapenem sparing. Antibiotic stewardship and strict infection control measures should be applied to limit the spread of these pathogens.

The limitations of our study include the relatively small sample size, so further studies with a larger sample size are warranted to confirm these findings. Additionally, it is important to assess the effectiveness of cefepime-tazobactam combination on patients through *in-vivo* studies.

## Data Availability

No datasets were generated or analysed during the current study.
